# Efficiency of clear aligners with nickel titanium wires for treating mandibular incisor crowding

**DOI:** 10.6026/973206300200917

**Published:** 2024-08-31

**Authors:** Yohan Verghese, Khumanthem Savana, Bhumika Khattar, Omar Basheer Altaher Mohammed, Prerna Raje Batham, Ulrika Diana Pereira Kalia, Noura Alessa

**Affiliations:** 1 Department of Orthodontics, Saveetha Dental College, Chennai, Tamil Nadu, India;; 2 Department of Orthodontics and Dentofacial Orthopedics, Dental College Regional Institute of Medical Sciences, Imphal, Manipur, India;; 3 Department of Orthodontics and Dentofacial Orthopedics, JN Kapoor DAV (c) Dental College, Yamuna Nagar, Haryana, India;; 4 Sebha Dental Faculty, Sabha University, Libya;; 5 Department of Orthodontics and Dentofacial Orthopedics, Government College of Dentistry, Indore, MP, India;; 6 Department of Orthodontics and Dentofacial Orthopedics, Bhabha College of Dental Sciences, Bhopal, MP, India;; 7 Department of Paediatric dentistry and Orthodontics, Dental College, King Saud University, Riyadh, Saudi Arabia;

**Keywords:** Anterior crowding, clear aligners, nickel-titanium

## Abstract

Patients who are allergic to nickel, which is present in stainless steel brackets and wires, commonly utilise clear aligners. The objective of this research was to compare the management of mandibular incisor crowding with nickel titanium (NiTi) wires and clear aligners. A random division of forty participants into two groups of twenty samples each was made. Participants in group B received transparent aligners, whereas those in group A received NiTi arch wires. We assessed malocclusion using Little's irregularity index. A survey instrument was utilised to document the degree of discomfort. Patients were routinely recalled every one, two, and three months. The mean score for groups A and B was 2.86 mm and 2.88 mm at baseline, 1.71 mm and 1.52 mm at one month, 1.02 mm and 1.23 mm at two months, and 0.72 mm and 0.48 mm at three months, respectively. The difference wasn't that big (p > 0.05). In groups A and B, the mean change in Little's irregularity index score was 1.19 mm and 1.21 mm at one month, 0.55 mm and 0.51 mm at two months, and 0.26 mm and 0.45 mm at three months, respectively. The difference wasn't that big (p > 0.05). For groups A and B, the corresponding mean discomfort scores were 2.6 and 2.3 at baseline, 2.2 and 1.8 after one month, 1.6 and 1.5 at two months, and 1.1 and 0.9 at three months. Since p > 0.05, the difference was not significant. The results of this study showed that NiTi wires and clear aligners worked just as well to treat cases of mandibular anterior crowding.

## Background:

The treatment of malocclusions has made great use of fixed orthodontics. Even though fixed orthodontic appliances have formed the foundation of orthodontic biomechanical approach, people still refuse to wear braces because they are unsightly [1]. Even though adults are almost as often as children and adolescents to develop malocclusions, they typically refuse to wear orthodontic wires, bands, and brackets because of discomfort or pain associated with the procedure [2]. The most frequent usage of nickel titanium wires in daily labial method practice is for tooth alignment. Super-elasticity, torsional strength, physiological compatibility, stress constancy, and shape memory are some of these wires' benefits [1]. When there is crowding of the lower anterior teeth, nickel titanium (NiTi) wires are recommended. These reduce time and are far more efficient than stainless steel wires [3]. Superelastic NiTi wires have greater torsional strength and stress constancy. Their wear resistance hysteresis, physiological shape, memory, compatibility, and dynamic interference are superior to those of other wires. These wires are helpful in shorter inter bracket spans, like for mandibular lower incisors, because of all these characteristics [4].

The aesthetic drawback of traditional fixed orthodontic treatment using orthodontic steel wire made of nickel or stainless steel is present. In the current field of orthodontics, a number of novel procedures have been created to improve patient comfort and aesthetics [1]. As a result of recent advancements in orthodontics, patients are increasingly choosing aesthetic orthodontic appliances, including as clear aligners, plastic brackets, ceramic brackets, lingual appliances, and aesthetic coated arch wires [1, 2, 5].

Recent developments include the widespread use of clear aligners for individuals allergic to nickel, which is present in stainless steel brackets and wires. In the world of orthodontics, clear aligners are the technology that is expanding the fastest [6]. Clear aligners may be recommended in situations of mild to moderate malposition, spacing, constricted non-skeletal arches, and relapsed patients following fixed appliance therapy. Because patients can readily take out these aligners on their own, there is less of an oral hygiene requirement [1, 7]. Additionally, clear aligners are a suitable option for people who have nickel allergy [1]. Shorter treatment times and segmental tooth movement were two benefits of clear aligners [8]. The objective of this research was to compare the management of mandibular incisor crowding with nickel titanium (NiTi) wires and clear aligners.

## Materials & Method:

After receiving approval from the institutional ethical committee, current prospective cross-sectional clinical study was carried out in the orthodontics department. Written consent was acquired from each patient, and they were all told about the study. The formula n= [(zα+zβ)] was used to get the sample size. A sample size of 40 was decided for the study, with a 95% confidence interval and 95% power. A sample size of at least 18 is thought to be sufficient for a P value of less than 0.05. Therefore, 20 samples from each group were taken into consideration for significance in the current investigation. Adult patients of both genders who were over 20 years old and had mild to moderate mandibular anterior crowding met the inclusion criteria; patients with poor periodontal health, prosthetic lower anterior teeth replacement, skeletal irregularities, and non-consent were excluded.

Every patient's demographic information was documented. Casts were created and dental impressions taken. In addition to the oral radiographs, photos and cephalogram as well as panoramic radiographs were taken. An individual not affiliated with the study randomly and equally separated 40 patients with mandibular anterior crowding of both genders into two groups, each consisting of 20 samples. Group B patients received transparent aligners, while Group A patients received the same initial NiTi arch wires for all participants. With the use of Little's irregularity index, crowding was evaluated. The overall irregularity score is calculated by adding the linear horizontal linear dislocation of the anatomic contact points of the mandibular anterior teeth. For the study, a calliper was employed, and millimetre measurements were made. Measuring zero denotes excellent alignment, three nominal irregularities, four to six moderate irregularities, seven to nine severe malposition's, and ten very severe irregularities. A 7-point Likert scale, with 5 representing no pain and 75 representing the worst agony, was employed in the questionnaire to measure the participants' levels of discomfort. Patients were routinely called back after one, two, and three months to assess the success of the two surgeries. One skilled investigator completed the entire process. The mean ± SD (mm) results were presented, and an ANOVA test with p <0.05 was used for statistical analysis using IBM's Statistical Package for the Social Sciences (SPSS) version 23.0 of Chicago, USA.

## Results:

According to Table 1, the mean score for groups A and B were 2.86 mm and 2.88 mm at baseline, 1.71 mm and 1.52 mm at one month, 1.02 mm and 1.23 mm at two months, and 0.72 mm and 0.48 mm at three months, respectively. The difference wasn't that big (p > 0.05). Table 2 demonstrates that in groups A and B, the mean change in Little's irregularity index score was 1.19 mm and 1.21 mm at one month, 0.55 mm and 0.51 mm at two months, and 0.26 mm and 0.45 mm at three months, respectively. The difference wasn't that big (p > 0.05). Table 3 demonstrates that for groups A and B, the corresponding mean discomfort scores were 2.6 and 2.3 at baseline, 2.2 and 1.8 after one month, 1.6 and 1.5 at two months, and 1.1 and 0.9 at three months. Since p > 0.05, the difference was not significant.

## Discussion:

The range of aesthetically pleasing orthodontic appliances available to patients has expanded due to recent advancements in orthodontics [1]. Clear aligners have been used to successfully repair malocclusions ranging from mild to severe. The benefits of clear aligners include improved comfort, dental hygiene, and aesthetics [2]. In order to treat mandibular incisor crowding, NiTi wires and clear aligners were examined in this study. Although the difference was not statistically significant, we discovered that using a clear aligner caused far less discomfort than using NiTi wires. In the current study, both groups' Little's irregularity index scores decreased from baseline to three months of treatment. When managing mandibular incisor crowding, Ashutosh *et al.* compared transparent aligners and nickel titanium (NiTi) wires. They came to the conclusion that mandibular anterior crowding situations may be managed just as well using NiTi wires and clear aligners [2]. The use of nickel titanium wires and transparent aligners for treating mandibular incisor crowding was compared by Melethil *et al.* They came to the conclusion that mandibular anterior crowding could be effectively managed with both nickel titanium wires and clear aligners [5]. In the lower anterior region, Bhatia *et al.* examined the aligning efficacy of two modalities: fixed appliances with nickel titanium wires and clear aligner therapy. According to the study, lower anterior crowding can be resolved with clear aligners as well as traditional fixed therapy [1]. These days, clear aligners are becoming more and more popular, and tests have demonstrated that they work just as well as NiTi wire [9, 10]. During the first year of therapy, the OHRQoL of patients receiving clear aligners is not as affected as that of patients receiving conventional fixed appliances [6].

To find out if the treatment efficacy of clear aligners was comparable to that of traditional fixed appliances, Ke *et al.* conducted a systematic review. They discovered that braces and clear aligners worked well together to cure malocclusion [11]. The effectiveness and efficiency of treating adolescents with Class I and II moderate to severe malocclusions using clear aligners (CAT) versus fixed appliances (FAT) was evaluated by Choua *et al.* They discovered no discernible difference in the effectiveness of therapy between fixed orthodontic appliances and clear aligners [12]. According to Borda *et al.*, clear aligners were just as successful as fixed appliances for treating minor malocclusions in teenagers. Treatment with transparent aligners produced greater results overall over the course of treatment [13]. According to Al-Sabbagh *et al.*, patients experienced less discomfort and a shorter treatment period using clear aligners as opposed to fixed therapy [14]. Clear aligners have been proposed by Al Mogbel *et al.* as a potential useful substitute for traditional braces in their systematic study [15]. There is a common misconception that transparent aligners can only tip crowns rather than roots because to the lack of control over tooth movement [15]. After using Smart Track® aligners, Eissa *et al.* assessed the root lengths of the upper incisors as a measure of the extent of apical root resorption caused by orthodontic therapy. They came to the conclusion that using Smart Track® aligners reduced root resorption in comparison to conventional fixed appliances [16]. The current study's limitations include a smaller sample size and the absence of wire comparisons. It is necessary to do additional study using bigger sample sizes in other regions.

## Conclusion:

Clear aligners can be helpful for patients who have aesthetic issues. The results of this study indicate that NiTi wires and clear aligners both worked just as well to treat cases of mandibular anterior crowding. In orthodontics, clear aligners are a recent development that may help patients who are more concerned with appearance.

## Financial support and sponsorship:

Nil.

## Figures and Tables

**Table 1:** Assessment of Little's irregularity index in both groups at various time intervals

**Time interval**	**Group A (NiTi)**	**Group B (Aligner)**	**P**
Baseline	2.86	2.88	0.5
1 month	1.71	1.52	0.9
2 months	1.02	1.23	0.1
3 months	0.72	0.48	0.1
P>0.05, non-significant

**Table 2:** Little's irregularity index score variation in both groups during various time periods

**Time interval**	**Group A**	**Group B**	**P**
1 month	1.19	1.21	0.8
2 months	0.55	0.51	0.1
3 months	0.26	0.45	0
P>0.05, non-significant

**Table 3:** Scores for discomfort in both groups at various intervals of time

**Time interval**	**Group A**	**Group B**	**p**
Baseline	2.6	2.3	0.6
1 month	2.2	1.8	0.8
2 months	1.6	1.5	0.9
3 months	1.1	0.9	0.9
P>0.05, no significant
